# A fast and effective approach for reversible wetting-dewetting transitions on ZnO nanowires

**DOI:** 10.1038/srep35073

**Published:** 2016-10-07

**Authors:** Kavita Yadav, B. R. Mehta, Saswata Bhattacharya, J. P. Singh

**Affiliations:** 1 Department of Physics, Indian Institute of Technology Delhi, Hauz Khas, New Delhi-110016, India

## Abstract

Here, we demonstrate a facile approach for the preparation of ZnO nanowires (NWs) with tunable surface wettability that can be manipulated reversibly in a controlled manner from a superhydrophilic state to a superhydrophobic state. The as-synthesized ZnO NWs obtained by a chemical vapor deposition method are superhydrophilic with a contact angle (CA) value of ~0°. After H_2_ gas annealing at 300 °C for 90 minutes, ZnO NWs display superhydrophobic behavior with a roll-off angle less than 5°. However, O_2_ gas annealing converts these superhydrophobic ZnO NWs into a superhydrophilic state. For switching from superhydrophobic to superhydrophilic state and vice versa in cyclic manner, H_2_ and O_2_ gas annealing treatment was used, respectively. A model based on density functional theory indicates that the oxygen-related defects are responsible for CA switching. The water resistant properties of the ZnO NWs coating is found to be durable and can be applied to a variety of substrates including glass, metals, semiconductors, paper and even flexible polymers.

Recently, smart surfaces with controlled reversible and switchable wettability by external stimuli have attracted significant attention due to their potential applications including self-cleaning surfaces, inkjet printing, microfluidic channels, and controllable drug delivery^1^,^2^,^3^. Generally in these applications, the superhydrophilic to superhydrophobic reversible switching is highly preferred. Many strategies have been put forward by researchers to achieve this reversible wettability switching by using external stimuli such as; light illumination^4^,^5^,^6^,^7^,^8^, temperature treatment^9^,^10^,^11^,^12^, pH control^13^, surface roughness^14^,^15^,^16^,^17^ and/or chemical modification^18^,^19^,^20^,^21^,^22^. However, all of the above mentioned approaches are either less effective or follow complex procedure. For example, the light illumination approach requires a long time, even several weeks, which makes the switching process less useful. Hence, for practical and real life applications, it would be highly desirable to accomplish the wettability transition by a convenient and time-saving method. Along with the reversible wettability transition, the superhydrophobic and superhydrophilic states, by themselves, are a topic of interest in both academics and industry because of their vast applications^23^.

In the past few decades, the wetting properties of metal-oxide-nanostructured surfaces have been widely studied. As a wide band gap semiconductor, ZnO possesses good transparency and high electron mobility, which make it suitable for a wide range of technological applications. Lee and co-workers have shown that electronic devices based on superhydrophobic ZnO nanorods overcome water vulnerability effects^24^. Superhydrophilic ZnO nanostructures show enhanced chemical and biological sensing^25^. These interesting features have stimulated extensive research in the design of ZnO nanostructures with superhydrophobic and superhydrophilic surfaces. Practical approaches in the preparation of superhydrophobic ZnO nanostructures include the alteration of surface roughness or surface energy modification. Surface roughness modification includes surface patterning^26^, or micro/nano structuring^27^ which includes strict growth conditions and complex processing techniques. However, the ZnO surface energy modification include low surface energy chemical coatings to enhance ZnO hydrophobicity^24^,^28^,^29^,^30^, which involves harsh chemical treatment. Moreover, the extent to which such approaches are effective depends upon the nature of the surface material. Thus, smart approaches are required to overcome these limitations.

This work presents a novel approach for fabricating superhydrophobic ZnO nanowires (NWs) and their reversible water wettability switching from a superhydrophilic to a superhydrophobic state. The surface wettability is shown to be controlled by simply tuning the oxygen-related defects. The ZnO NWs can be made superhydrophobic/superhydrophilic by H_2_/O_2_ gas annealing treatment, and reversible wettability switching is also possible. These superhydrophobic coatings of ZnO NWs can be applied to various types of substrates on which direct ZnO NWs growth via chemical vapor deposition (CVD) is not possible due to high growth temperature.

## Results and Discussion

**Figure 1:**
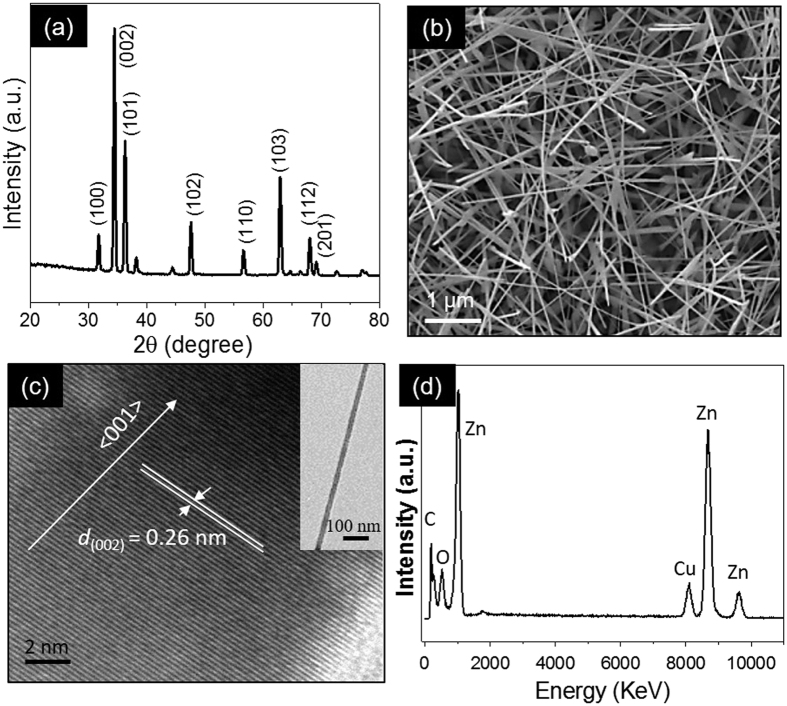
(**a**) GAXRD spectrum and (**b**) SEM image of the as-synthesized material. (**c**) The HRTEM image reveals the growth of a ZnO nanowire along the <001> direction and the inset is a TEM image of a single nanowire. (**d**) point EDX acquired from the single nanowire shows that the NWs are ZnO. The observed Cu signal in the EDX are from the TEM grid.

The ZnO NWs were grown via chemical vapor deposition (CVD). Figure 1a presents a glancing angle X-ray diffraction (GAXRD) spectrum which reveals that the ZnO nanostructures have a hexagonal wurtzite structure with lattice constant *a* = 0.325 nm and *c* = 0.521 nm. The scanning electron microscopy (SEM) image of as-grown ZnO samples is shown in Fig. 1b. The sample contains a nanowire-like morphology with a diameter of about 80 to 110 nm. The crystalline nature and composition of the ZnO NWs were investigated by transmission electron microscopic (TEM) studies. The bright field TEM image of a single nanowire is shown in inset of Fig. 1c. The high resolution transmission electron microscope (HRTEM) analysis reveals that the nanowires are crystalline. The interplanar spacing value of *d* = 0.26 nm corresponds to the (002) plane, which indicates the nanowires grow along the <001> direction. The point energy dispersive X-ray (EDX) pattern (Fig. 1d) acquired from a single nanowire confirms that the NWs are of ZnO. The Cu signal observed in the EDX are from the TEM grid.

The surface wettability of the samples was analyzed by measuring the average static contact angle (CA) for a sessile water droplet of 5 μl. The water droplet spreads completely on the as-synthesized ZnO NWs samples and the surface displays superhydrophilic behavior with a CA value of 0°. When these ZnO NWs samples were stored in the dark for about 50 days, the surface wettability changes drastically and the samples become superhydrophobic with a CA value of 155.7°. For reversible wettability switching, many reports in the literature have focused particularly on the light illumination method^31^,^32^,^33^. We have performed photoinduced wettability switching measurements on our ZnO NWs and the results are shown in the supplementary information, Fig. S1. An ultra violet (UV) illumination treatment with a Phillips UV lamp (11 watt) for 15 min is sufficient to convert the superhydrophobic ZnO NWs into a superhydrophilic state. The ZnO NWs show similar reversible wetting behavior with alternative dark storage and UV-illumination treatments as seen in the literature reports^31^,^32^,^33^. It is important to note that the dark storage method utilized to achieve superhydrophobic states is a very long process. It takes a few weeks’ time of dark storage to change the superhydrophilic state of as-synthesized ZnO NWs to a superhydrophobic state. This may not be acceptable for the realization of many practical applications.

**Figure 2:**
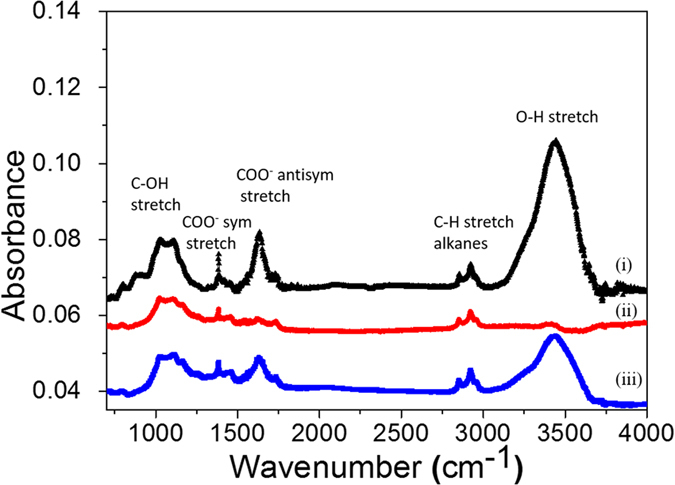
FTIR spectra of (i) as-synthesized, (ii) dark storage and (iii) UV treated ZnO NWs samples.

To develop a smart approach for preparing superhydrophobic ZnO NWs rapidly and economically without using any chemical coating, firstly we have to understand the water wetting mechanism on ZnO NWs. The water wetting of metal oxide surfaces is generally governed by the surface topography and the surface defects. In the present case, a nanowire-like morphology was used for water wetting measurements and the same type of samples show both superhydrophilic and superhydrophobic behavior under UV light illumination and dark storage. Hence, the surface morphology doesn’t play significant role in the observed wide variation of CA on ZnO NWs samples. A comparative defect analysis of three types of ZnO nanowire samples have been performed to analyze the defects responsible for the variation in the water wetting behavior with dark storage and UV treatments. The ZnO NWs samples used for this analysis were as-synthesized ZnO NWs (superhydrophilic), dark storage (50 days) ZnO NWs (superhydrophobic) and UV-treated (15 min) ZnO NWs (superhydrophilic). Fourier transform infrared spectroscopy (FTIR) was used to examine the defects radicals attached on the surface of the ZnO NWs. The FTIR measurements were conducted using a Thermo Scientific Nicolet^TM^iS^TM^50 system. These spectra were recorded in transmittance mode, in which ZnO NWs were scratched from the Si substrate and mixed with KBr to form a pellet. A series of absorption peaks from 600 to 4000 cm^−1^ are found corresponding to vibrations of various impurities such as hydroxyl (*-OH*), carboxylate (*COO*^*−*^) and alkane (*C-H*) present on the ZnO NWs surfaces. The FTIR spectra of these three samples are shown in Fig. 2. The most intense broad peak was observed at 3440 cm^−1^ assigned to hydroxyls for both dissociated and molecularly adsorbed water. Peaks between 2830 and 3000 cm^−1^ are due to the *C-H* stretching vibration of alkane groups. The peaks observed at 1630 cm^−1^and 1384 cm^−1^ are due to asymmetrical and symmetrical stretching of carboxylate groups (*COO*^*−*^), respectively^34^,^35^,^36^. The FTIR results indicate that the density of hydroxyl and carboxylate groups are maximal in as-synthesized ZnO NWs samples but decreases and almost disappears in the dark stored samples. This may be because of their thermodynamic instability. The hydroxyl-terminated surfaces show comparatively higher water wetting^37^,^38^. The significant change in the water contact angle in the three samples is consistent with the hydroxyl defects concentration.

**Figure 3:**
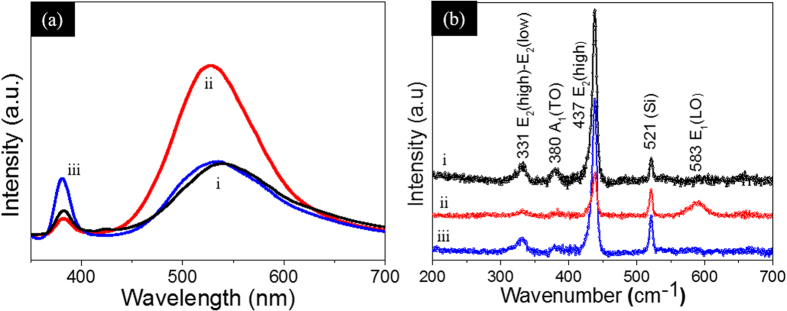
(**a,b**) Photoluminescence and Raman spectra of (i) as-synthesized, (ii) dark storage and (iii) UV-treated ZnO NWs samples.

Photoluminescence (PL) and Raman measurements were done on these samples to analyze the possible defects present in these ZnO NWs samples. For PL measurements, a Horiba Jobin Yvon Lab RAM (HR 800 Evolution) system was used with a He–Cd laser of 325 nm wavelength and 30 mW power as an excitation source. The room temperature PL spectra is shown in Fig. 3(a). The as-synthesized ZnO NWs samples show two different PL peaks positioned at 380 nm and 525 nm. The UV peak at 380 nm is ascribed to the near band edge emission^39^. The visible green emission is attributed to oxygen vacancy defects^40^,^41^. For the dark storage samples, the intensity of green emission increased and the UV emission decreased. This may suggest that the process of dark storage increases the oxygen vacancy defects in ZnO NWs. Similar results were reported in the literature where visible emission was enhanced with the aging process of ZnO nanowires^42^. However, the UV illuminated samples show reduced green emission. This is because the UV light generates electrons which react with surface oxygen to form 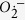 whereas the oxidation of water (or 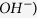 by holes produces the hydroxyl radicals (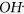) on the surfaces of ZnO NWs. The adsorption of oxygen onto the ZnO NWs surface passivates the oxygen vacancy centers and hence results in reduced green PL emission. The UV PL peak intensity is enhanced upon UV illumination due to the passivation of deep level defects.

The Raman measurements were acquired by using a Horiba Jobin Yvon Lab RAM (HR 800 Evolution) system with an Ar-ion laser of 514 nm wavelength and 50 mW power. The Raman spectra of the as-synthesized, dark stored and UV-treated ZnO NWs samples are shown in Fig. 3(b). All peaks in the Raman spectra can be assigned to the ZnO wurtzite structure^43^. The peak at 331 cm^−1^ corresponds to the second order Raman spectrum arising due to the E_2_ (high)-E_1_ (low) multiple scattering process of ZnO, 380 cm^−1^ corresponds to A_1_ (TO) and the main peak at 437 cm^−1^ is assigned to the E_2_ (high frequency) optical phonon mode of ZnO which is associated to oxygen atoms present in the ZnO^44^. The E_1_ (LO) mode at 583 cm^−1^ is associated with lattice defects as oxygen vacancies and zinc interstitials^45^,^46^. The high intensity of the 437 cm^−1^ peak and the absence of the 583 cm^−1^ peak indicates the oxygen rich nature of the ZnO NWs in the as-synthesized and UV treated ZnO NWs samples, whereas the enhanced 583 cm^−1^ peak in dark storage sample indicates its oxygen deficient nature. The defects observed in the Raman spectra are in consistent with the PL results and suggest that the dark storage ZnO samples are relatively oxygen deficient compared to the as-synthesized and UV-light illuminated ZnO NWs samples. A comparative analysis of CA and oxygen-related defects on the ZnO NWs reveal that the ZnO NWs surface with rare oxygen vacancy defects is more favorable for water wetting as compared to oxygen deficient ZnO NWs surface.

A first-principles based density functional theory (DFT) calculation was performed to understand the water wetting phenomenon on different types of ZnO NWs surfaces. The nanowires are randomly oriented, and hence, it is quite possible that different types of planes come into contact with the water droplet during contact angle measurements. The ZnO nanowires show the wurtzite crystal structure where generally four low miller index surfaces dominates, i.e., the nonpolar (10
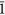
0) and (11
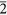
0) surfaces and the polar zinc terminated (0001)-Zn and oxygen terminated (000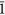)-O surfaces^47^. It was reported earlier that the nonpolar surfaces show surface relaxation with the tilt of the surface cations-anions dimers only. But, the polar surfaces stabilize via massive surface reconstruction or exhibit faceting to accommodate the charge transfer. Also, the polar surface stabilization may include randomly distributed vacancies, impurity atoms or charged adsorbates on the surface layers. The charged adsorbates and defects on the nanowire surface strongly affect the water wetting properties of the nanostructures. The DFT calculations for three different cases viz. (a) clean, (b) hydrogen passivated and (c) oxygen deficient (000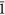) surfaces of ZnO were performed using FHI-aims code^48^, which is an all-electron code. The exchange and correlation functionals were used respectively (a) the generalized gradient approximations (GGA) as in perdew-Burke-Ernzerhof (PBE) implementation^49^ for geometry relaxation and (b) the more advanced hybrid functional as described in HSE06^50^ is used for total energy calculations. The later is used for estimation of the surface free energy of different cases. The *k*-mesh was generated by the Monkhorst−Pack method, and all results were tested for convergence with respect to the mesh size (6 × 6 × 1). In all calculations, self-consistency was achieved with a 0.1 meV convergence of the total energy and the force was minimized using a BFGS scheme up to 0.01 meV/Å tolerance value. Van der Waals corrections were taken into account as per the Tkatchenko−Scheffler scheme^51^.

The surface free energy is defined as


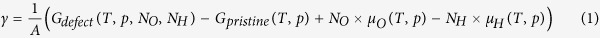


Where *G*_*defect*_ and *G*_*pristine*_ are respectively the Gibbs’ free energy of formation of the systems with and without defects, *μ*_*O*_ and *μ*_*H*_ are the chemical potentials of the ligands viz. O_2_ and H_2_ respectively. *N*_*O*_ and *N*_*H*_ are the number of O-vacancies and adsorbed H-atoms on top of the surface and *A* is the total area of the top surface. We have noticed that the surface free energy of the O-terminated plane is greater than the Zn-terminated plane. Therefore, we have considered the O-terminated 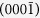 ZnO plane as our model system. All the surfaces in our study were modelled in terms of supercells of suitable dimension in the *x* and *y*-directions, and applied a vacuum layer of thickness 20 Å perpendicular to the surface.

**Figure 4:**
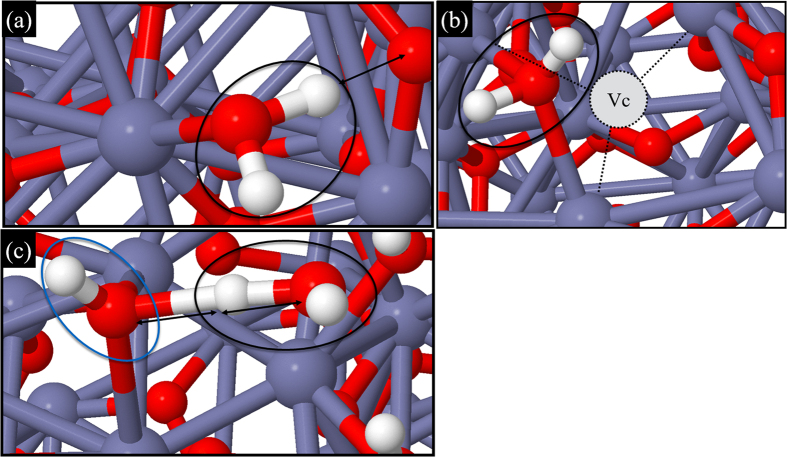
3D ball-and-stick model of the ZnO (0001) top surface with/without defects. The grey, red and white balls represent Zn, O, and H-atoms, respectively. (**a**) An H_2_O molecule is adsorbed on top of the Zn-site of the clean ZnO surface. (**b**) For the oxygen deficient ZnO surface, the H_2_O molecule is adsorbed near the O-vacancy site (Vc), (**c**) For ZnO (0001)-OH, the H_2_O molecule is dissociating from the surface. The H_2_O molecules adsorbed on the ZnO surface are encircled in black and the hydroxyl defect in blue. The double arrow represents the electrostatic force of attraction.

Next, we have compared the value of surface free energies of various cases in the O-terminated (000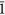) ZnO clean surface. We have modeled the clean surface and compared it with different types of neutral defects (viz. H-passivated or O-vacancy). The vibrational contribution to free energy calculations is expected to be small at low temperature and is therefore not taken into account. At a wide range of ambient conditions, the H-passivated surface has the highest surface free energy (e.g. temperature of 300 K and pO_2_=1atm and pH_2_=1atm) and is perfectly stable at 300 K. We have tried to understand the interaction of the surface with water molecules. It is noted that for clean and O-deficient surfaces the water molecule was adsorbed on top of the Zn-site (Fig. 4(a)) and O-vacancy (Fig. 4(b)) site, respectively, and no water dissociation was observed. However, when the water molecule was put on the OH-site (Fig. 4(c)), it dissociated. To understand the underlying interaction, we have estimated the Hirshfeld charge states of the respective atoms and found that O atoms are electron-rich, while Zn atoms are electron-deficient. On creating an O-vacancy, the electron of the O atom is shared equally amongst the three nearest neighbor Zn atoms. Therefore those three Zn-atoms become less electropositive but still sufficient to attract the lone pair electron of the O atom in H_2_O. Therefore, near the Zn-site (Fig. 4(a)) or O-vacancy site (Fig. 4(b)) H_2_O gets adsorbed. The electropositive H-atoms of H_2_O experience a feeble electrostatic force from the nearby electron-rich O atoms on the (000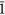) ZnO plane. But this force is not sufficiently large to break the O-H bond of the H_2_O molecule. Therefore, the H_2_O molecule remains stable on those sites making the surface superhydrophobic. On the other hand, on the H-passivated surface (surface with hydroxyl defects), there is a competition of sharing excess electron to the nearby-attached electropositive H-atoms between the ZnO surface O-atoms and the O-atom in H_2_O (see Fig. 4(c)).

There is a clear competition of reformation of bonds in the O-atom of the nearby hydroxyl groups that are attracted by the electropositive H-atom of the water molecule, at an instance when the H-atom experienced forces due to both O-atoms. A better representation is provided in a movie that is included in the supplementary information (Video V1). After studying all three cases we found that the as-synthesized ZnO NWs surface with a large number of hydroxyl defects are more favorable for water wetting because there is a higher probability of water dissociation. However, on the O-deficient surfaces, water wetting is minimal. These observations are in close agreement with our experimental findings.

**Figure 5:**
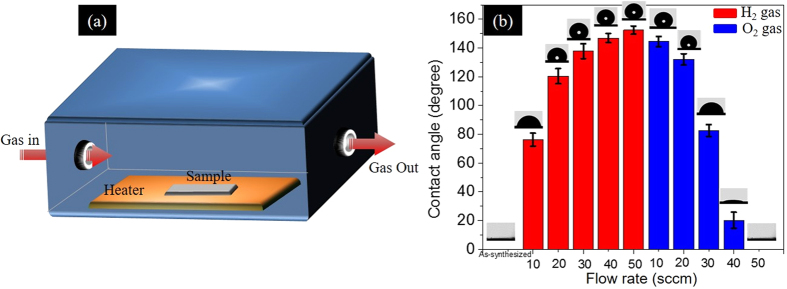
(**a**) Schematic of the chamber used for ZnO NWs sample annealing. (**b**) Tailoring of the contact angle of ZnO NWs by annealing in H_2_ and O_2_ gas atmospheres with different flow rates.

The temperature-dependent stability of hydroxyl defects on the surface of the O-terminated (000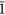) ZnO NWs surface was also analyzed. The complete details are mentioned in the supplementary information, section S2. The H-passivated surface is perfectly stable at 300 K but on increasing the temperature and keeping the pressure constant, we noticed that instead of having extra stability of the OH defects, the O-vacancies become predominant. This observation is in agreement with our free energy calculations. In view of this, our experimental findings showing more OH-defects on as-synthesized ZnO, strongly agree with our thermodynamic model calculations as well as molecular dynamics simulation. We have utilized the idea of removal of hydroxyl defects and the creation of surplus oxygen vacancy defects with annealing of the ZnO samples for controlling the water CA on ZnO NWs. Here, oxygen-related defects are tuned by using oxidizing (O_2_ gas) and reducing (H_2_ gas) ambient conditions during the annealing treatment of the ZnO nanowires. The ZnO NWs sample are treated at 300 °C in the presence of H_2_ or O_2_ gases. A series of experiments were done to calibrate the annealing parameters like H_2_/O_2_ gas flow rate and annealing time to obtain wettability switching of the ZnO NWs. The annealing temperature was kept constant at 300 °C to avoid the effect of the change in surface roughness at higher temperature^32^. The initial observations reveal that a 50 sccm flow rate of H_2_ gas at 300 °C temperature for 90 min is sufficient to make the ZnO NWs superhydrophobic with a CA of about 153.5°. The superhydrophobic ZnO NWs are highly water repellent as shown by supplementary video V2. The superhydrophobic ZnO NWs were then annealed in O_2_ gas at 300 °C for 60 min. Different flow rates of O_2_ gas were used for the annealing treatment and again a 50 sccm flow rate was found to be sufficient to regain the ZnO NWs superhydrophilic nature. The change in CA on the ZnO NWs with different annealing treatments is shown in Fig. 5. The H_2_ gas annealing treatment creates oxygen vacancy defects in metal oxides whereas the O_2_ gas annealing treatment passivates the oxygen vacancy defects^52^,^53^,^54^,^55^. Here, the change in oxygen vacancy defects after annealing treatment is analyzed by PL measurements and the results are shown in supplementary information, Fig. S3.

The heating treatment on the metal oxide nanostructures affects the contact angle^32^,^56^. To separate out the effect of heating on CA during the H_2_/O_2_ annealing, we have annealed the as-synthesized samples at 300 °C in Ar gas (50 sccm) for 60 and 90 min. The CA values found on 60 and 90 min Ar annealed ZnO NWs samples were 29° and 35°, respectively. The Ar gas was used to create an inert atmosphere, which should eliminate the effect of oxidizing (O_2_) and reducing (H_2_) gases present in the atmosphere. These observations suggest that water wetting on the ZnO NWs is strongly influenced by the presence of the H_2_/O_2_ gases during the annealing treatment.

**Figure 6:**
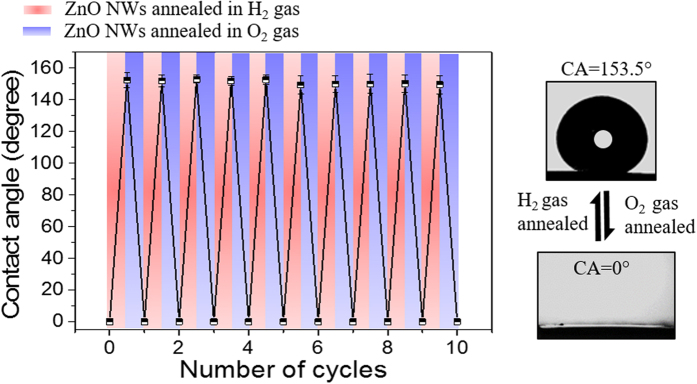
Reversible switching of ZnO NWs wettability from superhydrophilic to superhydrophobic state upon alternative H_2_ and O_2_ gas annealing treatment.

Further we found that the H_2_/O_2_ gas annealing treatments are not only suitable for tuning the contact angle but reversing the wettability of the ZnO NWs is also possible (Fig. 6). The as-synthesized ZnO NWs were superhydrophilic in nature. The H_2_ gas (50 sccm) annealing treatment at 300 °C for 90 min makes the samples superhydrophobic with average CA value of 153.5 °C and an O_2_ gas (50 sccm) annealing treatment at 300 °C for 60 min was observed to be sufficient to regain the superhydrophilic nature. Alternating the annealing treatment of the H_2_ and O_2_ gases produces superhydrophobic and superhydrophilic states of the ZnO nanowires, respectively. Up to 10 cycles, the annealing treatment seems to be suitable for completely reversible switching of the ZnO NWs between the superhydrophilic and superhydrophobic states. It is important to notice that the contact angle on superhydrophilic ZnO NWs samples is not stable. It increases with time and becomes hydrophobic (CA=123°) and superhydrophobic (CA=155.7°) when the samples are stored in daylight and under dark conditions, respectively (Fig. S4 in the supplementary information). But, the superhydrophobic samples become stable and retains their water repellent nature for more than 6 months.

We have also tested the H_2_/O_2_ gas annealing method for CA-tuning on other CVD grown nanostructures like indium oxide and gallium oxide NWs (see Fig. S5 in the supplementary information). The parameters used for the annealing treatment were the same as those used for the ZnO NWs. The observations are in accordance with the earlier findings: the H_2_ gas annealing treatment increases the hydrophobicity whereas the O_2_ gas annealing treatment increases the hydrophilicity of the metal oxide NWs. These results indicate that the H_2_/O_2_ gas annealing treatment is a fast and effective approach to control the demand-based water wetting behavior of different metal oxide nanowires.

**Figure 7:**
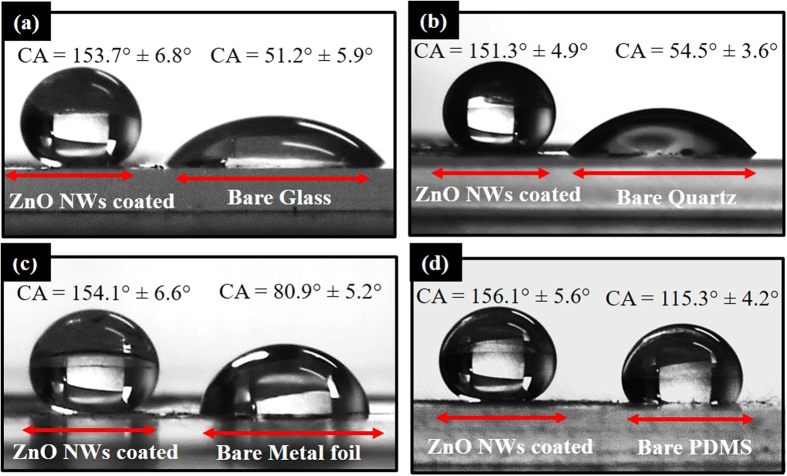
ZnO NW-coated superhydrophobic (**a**) glass, (**b**) quartz, (**c**) aluminum foil and (**d**) PDMS substrates.

Additionally, it was found that the superhydrophobic ZnO NWs can be utilized for hydrophobization of different types of substrates like glass, quartz, metals, semiconductors, paper and flexible polymers by dispersing ZnO nanowires onto them. The superhydrophobic ZnO NWs were scratched from the Si substrate and mixed into ethanol. The mixture was ultrasonicated for 15 min and then dispersed on different substrates by drop coating. Figure 7 shows CA images of a water droplet on different substrates where half part of the substrate is coated with superhydrophobic ZnO NWs. After the ZnO NWs coating, the water wetting behavior of various substrates show a drastic change, and the substrates become superhydrophobic. The transmittance measurements (see supplementary information Fig. S6) of superhydrophobic glass substrates show more than 80% transparency for visible light. The annealing treatment used in the present research work is simple. The annealing time (90 min) and temperature (300 °C) are conventionally used by the researchers and suit to the industrial scale. Moreover, these nanowires can be scratched and coated/dispersed on variety of substrates such as flexible polymer like polydimethylsiloxane (PDMS) to hard substrates like sapphire without losing their wetting properties.

In conclusion, we have accomplished the goal of preparing superhydrophobic ZnO NWs and controlling their reversible wettability switching from superhydrophilic to superhydrophobic states, by a fast and cost-effective approach. The ZnO NWs were grown by CVD. The as-synthesized ZnO NWs show a superhydrophilic nature and by an H_2_ gas (50 sccm) annealing treatment the ZnO NWs become superhydrophobic. However, the O_2_ gas (50 sccm) annealing treatment was observed to be suitable to revert the superhydrophilic nature of ZnO NWs. The controlled reversible wettability switching is demonstrated by the alternating annealing treatments of H_2_ and O_2_ gases. Also, it has been shown that the superhydrophobic ZnO NWs can be utilized for hydrophobization of different types of substrates. Furthermore, this novel approach might help to prepare superhydrophobic nanostructures of different metal oxides and their controlled reversible wettability switching for future applications.

## Methods

### Materials

ZnO powder (99.99%) was purchased from Sigma-Aldrich. Activated charcoal powder and ethanol were purchased from Merck Company.

### Fabrication of ZnO nanowires

The ZnO NWs were synthesized using horizontal a chemical vapor deposition (CVD) system. A mixture of ZnO and charcoal powder in a ratio of (1:1) was taken as a precursor and loaded into the alumina boat at the center of the tube furnace. Si substrates coated with ~5 nm gold were placed downstream in the tube furnace at a distance of 1 inch from the precursor boat. The furnace was then heated up to 1050 °C under a constant flow (200 sccm) of Ar gas. The system was at 1 atmospheric pressure.

### Characterizations

The samples were characterized using ZEISS EVO 50 scanning electron microscope (SEM), glancing angle X-ray diffraction (GAXRD) using phillips X’Pert, PRO-PW 3040 diffractometer and Technai G20-stwin high resolution transmission electron microscope (HRTEM) at 200 K. Photoluminescence (PL) and Raman measurements were taken using a Horiba Jobin Yvon Lab RAM (HR 800 Evolution) system. For PL measurements, 30 mW He–Cd laser of 325 nm wavelength was used as an excitation source. For the Raman measurements, an Ar-Ion laser of 514 nm wavelength and 50 mW power was used. The Fourier transform infrared (FTIR) measurements were done using a Thermo Scientific Nicolet^TM^iS^TM^50 system. UV-Vis transmittance measurements were done using a Shimadzu UV-3600. The wetting properties of the samples were studied by measuring the contact angle (CA) of a 5 μl water droplet on the samples at room temperature using an optical CA meter.

### Gas annealing treatment

A small chamber of dimensions 3 × 2.5 × 1.5 inch^3^ was fabricated for effective and controlled annealing of the ZnO NWs in different gaseous atmosphere. The gas flow rate was controlled by using an *Alicat Scientific* mass flow controller (MFC). A heater was used to heat the sample up to 300 °C during the annealing treatment.

## Additional Information

**How to cite this article**: Yadav, K. *et al*. A fast and effective approach for reversible wetting-dewetting transitions on ZnO nanowires. *Sci. Rep*. **6**, 35073; doi: 10.1038/srep35073 (2016).

## Supplementary Material

Supplementary Information

Supplementary Video S1

Supplementary Video S2
