# Supplementary material for: A fast and effective approach for reversible wetting-dewetting transitions on ZnO nanowires
Source: Sci Rep. 2016 Oct 7;6:35073. doi: 10.1038/srep35073 (PMC5054356; doi:10.1038/srep35073)
Supplement: Supplementary Information [file srep35073-s1.pdf]

Supplementary Information

A fast and effective approach for reversible wetting-dewetting transitions on ZnO nanowires

Kavita Yadav, B. R. Mehta, Saswata Bhattacharya, and J. P. Singh*

Department of Physics, Indian Institute of Technology Delhi, Hauz Khas, New Delhi-110016, India

* Corresponding author, Tel: 011-2659-1323, E-mail: jpsingh@physics.iitd.ac.in

Supplementary Videos

Video V1: This video shows the dissociation of the water molecule on the H passivated ZnO $(000\bar{1})$ plane. H_2O molecule is getting dissociated because of the competition of sharing excess electron to the nearby attached electropositive H-atoms between the ZnO surface O-atom and the H_2O O-atom on the H-passivated surface.

Video V2: This video shows the superhydrophobic and highly water repellent nature of ZnO NWs after H_2 gas (50 sccm) annealing treatment. If a water droplet of 5 μL is dropped on the superhydrophobic sample, the water droplet completely bounces on the sample surface.

Supplementary Figures

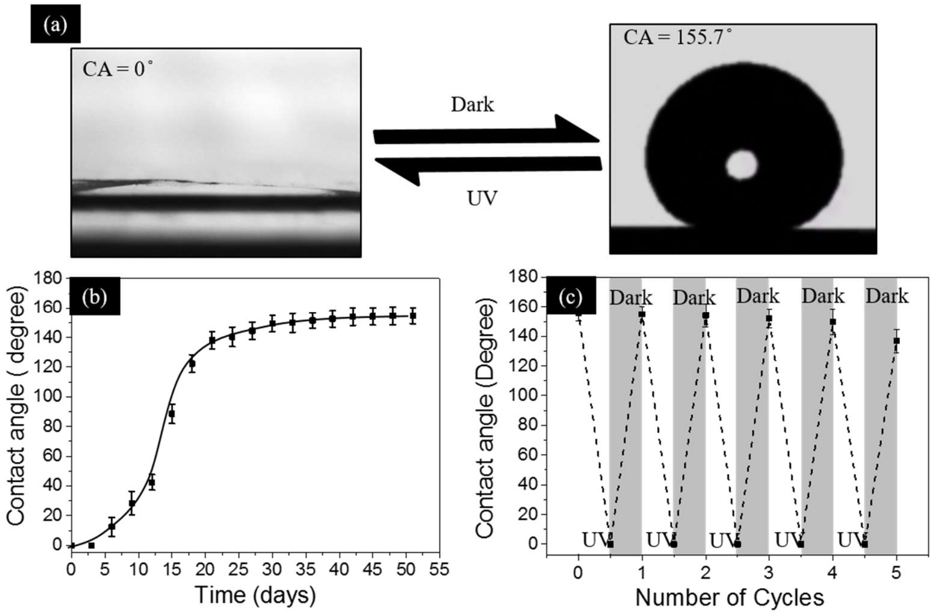

Figure S1. (a) Photoinduced reversible wetting of ZnO NWs, (b) recovery of contact angle on ZnO NWs with dark storage and (c) cyclic transition of CA from superhydrophobic to superhydrophilic state.

Section S2

We have analyzed the temperature dependent stability of hydroxyl defects on the surface of O-terminated $(000\bar{1})$ ZnO surface. At wide range of ambient conditions, the H-passivated surface is having the highest surface free energy (e.g. temperature of 300 K and pO_2=1atm and pH_2=1atm), while, the H-passivated surface is perfectly stable at 300 K. On increasing the temperature keeping pressure unchanged, O-vacancy starts becoming predominant. Note that our theoretical free energy calculation is valid for a situation under thermodynamic equilibrium only. Furthermore, at high temperature, vibrational contribution of the atoms plays a significant role in estimation of surface free energy γ. However, performing vibrational calculations of such a huge system consisting several hundreds of atoms is computationally quite expensive. Moreover, even if we have computed the explicit vibrational contribution to the free energy of formations using harmonic approximations, at high temperature due to anharmonic effects, the conventional procedure to compute the free energy of formation may not be sufficient. The configurational entropy is expected to play some role in the stability of the surface at high temperature. Therefore, in order to understand this extra stability of H-passivated top surface of the ZnO plane at higher temperature, we have performed 8ps molecular dynamics (MD) simulations at different temperatures (viz. 600 K, 800 K, 1200 K, etc.). We have used Nose hoover thermostat for performing NVT simulations at various temperatures. First we noted that H-passivated ZnO $(000\bar{1})$ plane is not stable at high temperature, as beyond 600 K (Figure S2), OH-defects are leaving the surface, creating surplus of O-vacancy sites. This observation is in agreement with our free energy calculations.

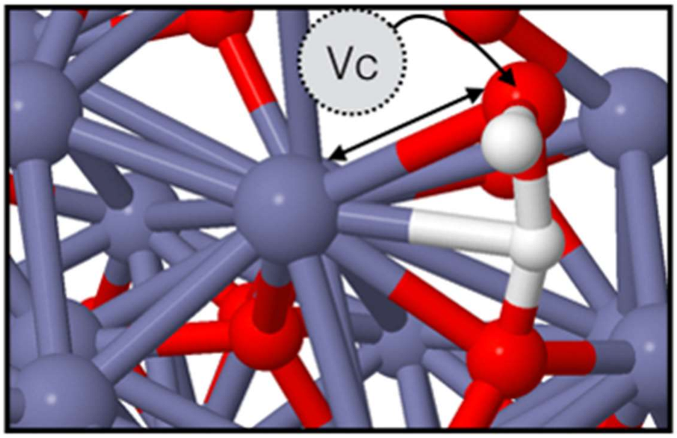

Figure S2. 3D ball and stick model structures of ZnO $(000\bar{1})$ top surface. Grey, red and white balls represent Zn, O, and H-atoms, respectively. 8ps MD snap-shot of ZnO $(000\bar{1})$-OH surface at 600 K. OH is leaving creating surplus of O-vacancy sites (Vc) on the surface.

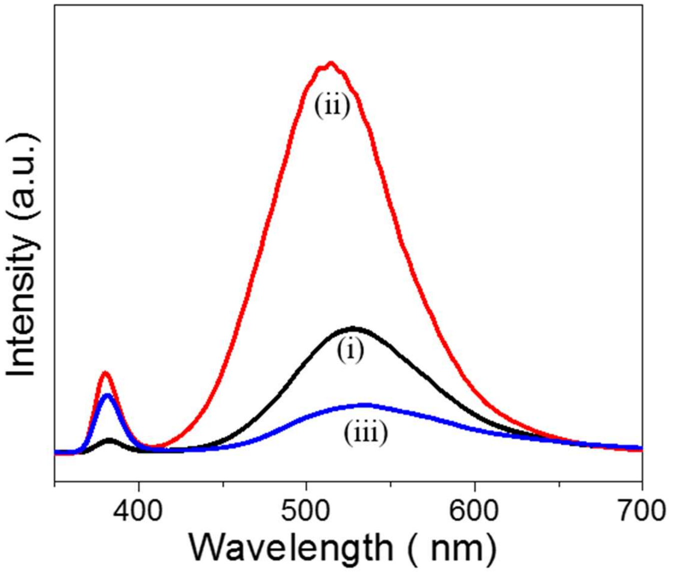

Figure S3. Room temperature PL spectra of (i) as-synthesized, (ii) H_2 annealed (50 sccm) and (iii) O_2 annealed (50 sccm) ZnO NWs.

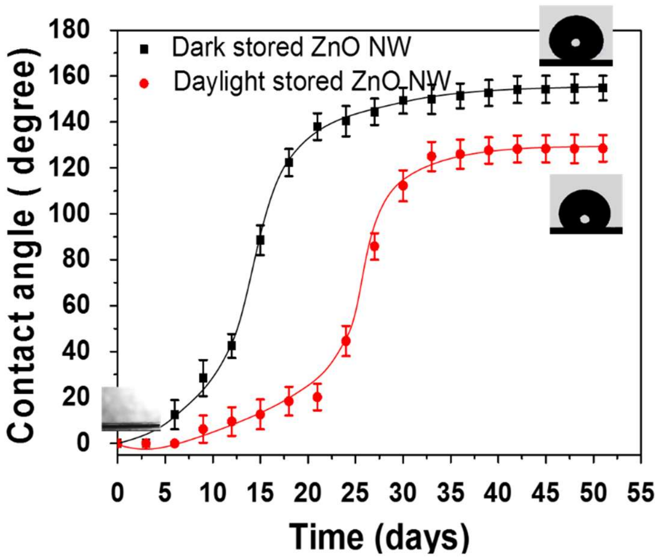

Figure S4. Transition of water contact angle in dark and day light stored ZnO nanowires sample with passage of time.

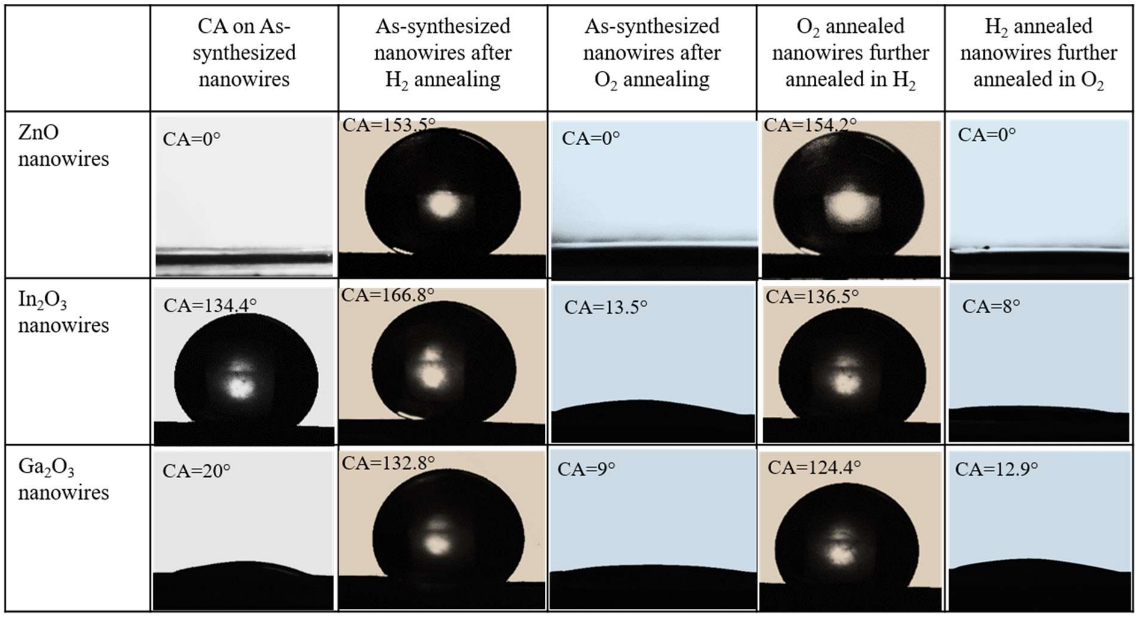

Figure S5. Variation of contact angle on zinc oxide, indium oxide and gallium oxide nanowires after H_2 and O_2 gas annealing treatment.

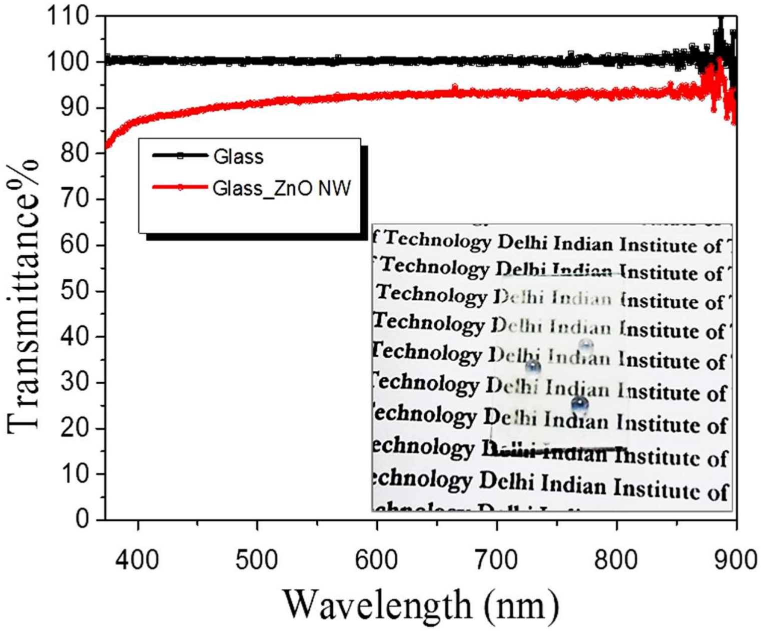

Figure S6. UV-Vis transmittance spectra of superhydrophobic ZnO nanowires coated on glass substrate. The optical image of superhydrophobic ZnO coated glass slide with water droplets is also shown.
